# A research agenda for scaling up agroecology in European countries

**DOI:** 10.1007/s13593-022-00786-4

**Published:** 2022-06-09

**Authors:** Chantal Gascuel-Odoux, Françoise Lescourret, Benoit Dedieu, Cécile Detang-Dessendre, Philippe Faverdin, Laurent Hazard, Isabelle Litrico-Chiarelli, Sandrine Petit, Lionel Roques, Xavier Reboud, Michele Tixier-Boichard, Hugo de Vries, Thierry Caquet

**Affiliations:** 1 Institut Agro, UMR SAS, INRAE, 35000 Rennes, France; 2 UR PSH, INRAE, 84000 Avignon, France; 3 ACT, INRAE, 63000 Clermont-Ferrand, France; 4 Institut Agro, UMR CESAER, INRAE, 21000 Dijon, France; 5 Institut Agro, UMR PEGASE, INRAE, 35000 Rennes, France; 6 INP-Toulouse, UMR AGIR, INRAE, 31000 Toulouse, France; 7 BAP, INRAE, 86600 Lusignan, France; 8 Institut Agro, UMR Agroécologie, INRAE, 21000 Dijon, France; 9 UR BIOSP, INRAE, 84000 Avignon, France; 10 Institut Agro, UMR Agroécologie, INRAE, 21000 Dijon, France; 11 AgroParisTech, UMR GABI, INRAE, 78352 Jouy-en-Josas, France; 12 Institut Agro, UMR IATE, INRAE, 34000 Montpellier, France; 13 Collège de direction, INRAE, 75238 Paris, France

**Keywords:** Agroecology, Agri-food systems, Territory, Research agenda, Collective prospective, Interdisciplinarity

## Abstract

A profound transformation of agricultural production methods has become unavoidable due to the increase in the world’s population, and environmental and climatic challenges. Agroecology is now recognized as a challenging model for agricultural systems, promoting their diversification and adaptation to environmental and socio-economic contexts, with consequences for the entire agri-food system and the development of rural and urban areas. Through a prospective exercise performed at a large interdisciplinary institute, INRAE, a research agenda for agroecology was built that filled a gap through its ambition and interdisciplinarity. It concerned six topics. For genetics, there is a need to study genetic aspects of complex systems (e.g., mixtures of genotypes) and to develop breeding methods for them. For landscapes, challenges lie in effects of heterogeneity at multiple scales, in multifunctionality and in the design of agroecological landscapes. Agricultural equipment and digital technologies show high potential for monitoring dynamics of agroecosystems. For modeling, challenges include approaches to complexity, consideration of spatial and temporal dimensions and representation of the cascade from cropping practices to ecosystem services. The agroecological transition of farms calls for modeling and observational approaches as well as for creating new design methods. Integration of agroecology into food systems raises the issues of product specificity, consumer behavior and organization of markets, standards and public policies. In addition, transversal priorities were identified: (i) generating sets of biological data, through research and participatory mechanisms, that are appropriate for designing agroecological systems and (ii) collecting and using coherent sets of data to enable assessment of vulnerability, resilience and risk in order to evaluate the performance of agroecological systems and to contribute to scaling up. The main lessons learned from this collective exercise can be useful for the entire scientific community engaged in research into agroecology.

## Contents

- 1. Introduction
- 2. Trajectory and methods for building a research agenda
- 3. Two goals defined at an early stage
- 3. 1 Redesigning agricultural systems all the way up to entire agri-food systems
- 3.2 Using ecological and socio-economic concepts for renewed research frameworks
- 4. Six issue-specific themes
  - 4.1 The genetics mechanism and its implications for animal and plant breeding
  - 4.2 The landscape mechanism to contribute to agroecosystem services
  - 4.3 Agricultural equipment and digital technology to better monitor biological systems
  - 4.4 Modeling to understand and predict dynamics of new agroecosystems
  - 4.5 The agroecological transition of the farm as a research topic
  - 4.6 Integration of agroecology into food systems
- 5. Issues common to all six themes and next steps
  - 5.1 From collecting biological data to using them to redesign agroecosystems in participatory mechanisms
  - 5. 2 Understanding adaptability and risks though a transformation process
  - 5.3 Scaling up
  - 5.4 Next steps: relevance of the research agenda, implementation and funding for agroecology
- 6. Conclusion
- Acknowledgments
- References

## Introduction

In industrialized countries, agricultural productivity and economic competitiveness have been improved by a modernization process that began in the 1950s. This modernization has taken the form of specialization of production systems; development of agricultural machinery; increased farm size; and increased use of water, chemical inputs, and plant varieties and animal breeds with high production potential. Advisory structures and technical references have been created to help the agricultural sector develop along these lines, and advice to farmers has aimed to optimize production through economic efficiency. This has led to homogenization of agricultural systems and to economic gains across the entire agri-food sector, ranging from agricultural production to standardized products for the agri-food industry (van der Ploeg et al. 2019). Agriculture has evolved by becoming increasingly industrialized, with an intensity that varies by region and sector, leading to mass-produced products of homogenous quality. Industrialization has generated externalities that were initially perceived as positive (e.g., “clean” environments without pests, high productivity). However, over time, an increasing number of negative externalities (e.g., soil, water and air pollution; greenhouse gas emissions; biodiversity loss) have been recognized (Campbell et al. 2017; Evans et al. 2019; Qiao et al. 2019; Sanchez-Bayo and Wyckhuys 2019; Stehle and Schulz 2015; Tsiafouli et al. 2015). In its global assessment report on biodiversity and ecosystem services, IPBES (2019) clearly identified land-use change and agricultural inputs as the main drivers of biodiversity loss.

A better compromise between agriculture and the environment has been sought over the past few decades through decreases in the use of inputs and improvements in their efficiency. Such optimization was nonetheless considered insufficient, and many emerging movements (e.g., organic farming, conservation farming, eco-farming) have developed concepts and terminology to better reconcile economic, social, environmental, and health dimensions (Garibaldi et al. 2017). In this context, agroecology appears as an essential path, because it is inclusive and based on general principles that put ecological processes at the heart of the redesign of a sustainable and resilient agriculture. Agroecology is not only for smallholder agriculture, in which it became most successful, but is urgently needed for large-scale farming (Tittonell et al. 2020). Agroecology has been defined in many ways, associating ecology to varying degrees with other disciplines (e.g., agronomy, genetics, sociology) and with local or traditional knowledge, and has aimed for sustainable production or even food systems by preserving and using biodiversity in agroecosystems (Wezel et al. 2018b).

Research institutions have gradually adopted agroecology as a structuring objective for all of their agricultural research. Consequently, agroecology is increasingly the subject of academic publications. The number of publications explicitly positioned on this disciplinary branch increased steadily from approximately 50 per year in 2005 to 350 per year at present, for a total of 13,632 over the past 25 years. This progress was recently analyzed (Mason et al. 2021). French sources are at third place (i.e., 8%) in this corpus of publications, after those from the USA and China (assessed in September 2020 using the query “agroecology OR agro-ecology OR agroecosystem* OR agro-ecosystem*” in the Web of Science, searching within titles, abstracts and keywords).

**Fig. 1:**
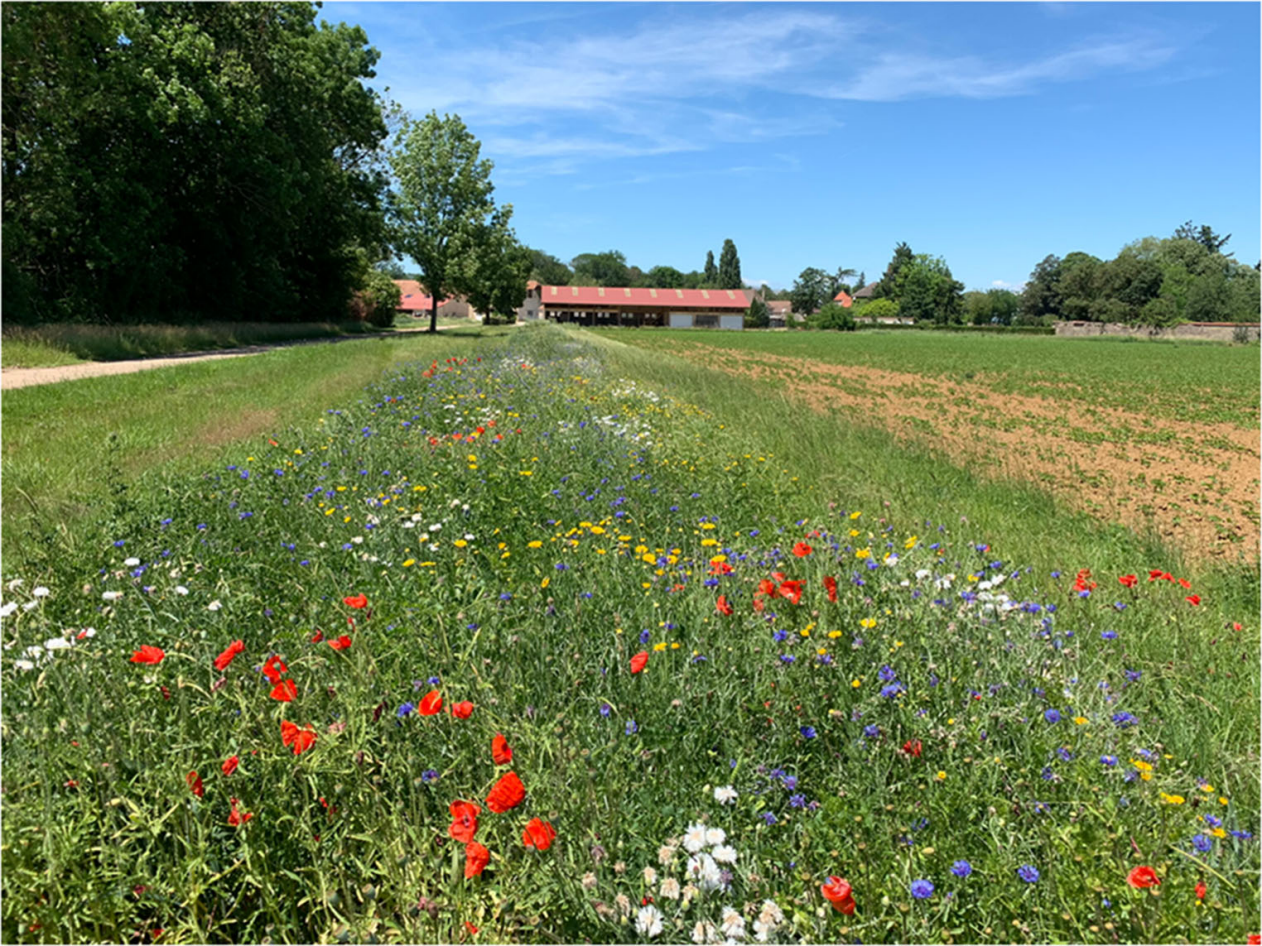
Experimental platform at landscape level (CA-SYS, INRAE), summer 2019. Photograph by Brice Mosa, INRAE.

INRAE, one of the largest agricultural research institutes in the world (with a permanent staff of 12,413) and well-known for its interdisciplinarity, accounts for 6% of this corpus of publications. As a research institute, it has taken a position on agroecology since 2011 (Figure 1) (INRA 2012; Soussana 2015) and is recognized for it (Wezel et al. 2018a). In 2017, it launched an interdisciplinary scientific prospective exercise to define the research topics to address in order to scale up agroecology in science for academic purposes as well as to help develop agroecology in society. A research agenda is a way to recognize and encourage, within INRAE and with its national and international research partners, major interdisciplinary research into agroecology. It also provides the opportunity to foster the appropriation of agroecological principles, obstacles, and mechanisms (e.g., Meynard et al. 2015) by a large scientific community. The results of this prospective exercise, which involved approximately 80 researchers, can be useful to the entire scientific community engaged in research into agroecology. They are especially relevant for countries with industrial agriculture, and thus for Europe, where industrial agriculture dominates to a greater or lesser degree within and among countries. In these countries, the transition of highly integrated agro-industrial systems is more difficult and wider in scope than the transition of a single sector of agricultural production.

This article first traces the trajectory of the inclusion of agroecology in the research agenda at INRAE, then presents the methodology and main results of the prospective exercise and the general lessons learned that will nurture future research programs. Finally, the discussion highlights ways in which other international and national scientific organizations, research-program managers and officers of research-funding bodies can take advantage of this exercise.

## Trajectory and methods for building a research agenda

In 2011, INRAE began a collective effort in which agroecology was considered as a new discipline at the interface between ecology and agronomy. During this initial stage (2011–2014), conferences by invited researchers, discussions with partners and actors, and internal discussions within INRAE were held, and a summary report was published for internal purposes (INRA 2012).

At the same time (2012), the French Ministry of Agriculture launched the “Agroecological project for France.” This convergence of the research and public policy agendas led most notably to the organization of a symposium in 2013, which brought together researchers, policymakers and actors from across the agricultural domain in France. The scientific portion of this symposium was published in a special issue of *Innovations Agronomiques*, introduced by Soussana (2015), with nine articles that addressed three major topics (i.e., adding biodiversity, managing landscapes and territories, and closing nutrient cycles) and issues (i.e., principles, current research, and examples developed in research and innovation with partners) to share the outputs of this symposium widely with the national community. For the first time, agroecology was included in the French public policy agenda, which encouraged combined efforts and interactions among a variety of actors. These national dynamics were in line with international dynamics driven by the Food and Agriculture Organization of the United Nations (FAO), which organized the 1st International Symposium on Agroecology for Food Security and Nutrition in 2014, with the goal of promoting agroecological systems at the international level, including in industrialized countries. This symposium was followed by a regional workshop on European agriculture.

In 2014, INRAE organized a workshop on contributions to and challenges for modeling agroecological systems as an essential approach to developing them (Garcia et al., 2014) in order to predict, manage, and evaluate them. This workshop also highlighted the role of actors and experimental facilities in building scenarios and evaluating agroecosystems. In 2015, scientific think-tank days were organized to address how to assess the multi-performance of agroecological systems, considering their multiple objectives, the many ways to transform them depending upon their context and the multiple dimensions of integration (i.e., space, time, environmental, social, and economic).

Consequently, in 2016, INRAE included in its orientation document *#Inra2025* (Vilotte et al. 2016) its decision to intensify and expand research into agroecology, as a co-fertilization between agronomy and ecology, with a transformative view of agricultural systems, all the way up to agri-food sectors and territories. Agroecology was no longer a simple and new way of looking at agronomy, but was part of a broader societal process, with economic, sociological, food, and environmental dimensions. In 2017, this decision was reflected in the launch of an interdisciplinary scientific prospective exercise that aimed to amplify research into agroecology across these dimensions. Six topics were defined at the highest level of INRAE governance: the role of (i) genetics and (ii) the landscape, both of which are often under-considered for agroecology; the role of (iii) agricultural equipment and digital technology and (iv) modeling, both of which use available information to monitor and redesign agroecological systems; and the agroecological transition (v) at the farm scale and (vi) of the entire agri-food sector, due to the urgent need to scale up agroecology.

The exercise was performed in four stages. First, a steering committee of 12 INRAE senior researchers discussed the relevance and the boundaries of the six topics, defined a common working method for developing each topic and monitored the progress of the collective work.

Second, six working groups, one for each topic, were formed. Each group was chaired by a member of the steering committee, and one or two senior researchers nominated by each of INRAE’s research divisions, to cover all disciplines of the institute. The groups held several meetings and used various data sources (e.g., literature, internal surveys, interviews) to identify knowledge gaps and requirements, draw research perspectives and identify relevant partnerships for future collaboration.

Third, two seminars were organized among the working groups to identify transversal research issues. Finally, the steering committee organized public presentation and discussion of the outcomes.

In this way, the approximately 80 researchers spent approximately 200 days involved in the exercise. This work has led to reports (Caquet et al. 2019), presentations, and a book (Caquet et al. 2020) to share the results among the research community, its partners and, even further, the national and international scientific and non-scientific community. The work was also presented and then discussed with actors at round tables, and presented at INRAE board meetings where actors were also present. This article describes only the research perspectives that had a general scope.

## Two goals defined at an early stage

### Redesigning agricultural systems all the way up to entire agri-food systems

Research may aim for the best possible use of resources, using the terms “smart agriculture” or “sustainable intensification”, which correspond to “weak” agroecology (Altieri et al. 2017; Saj et al. 2017). It advocates for continuity of current systems and does not explicitly call for biological processes to replace the use of inputs, but encourages a greater efficiency of the latter (Duru et al. 2015b). This weak agroecology, which includes many ideas found in “low-input” agriculture, contrasts with “strong” agroecology, defined by its goal of coherence and sustainability by using biological processes (Duru et al. 2015a, 2015b). Such leveraging of biological processes implies increased consideration for diversity in agroecosystems (Kremen et al. 2012) through aspects such as crop rotations, grassland and rangeland maintenance, selection and use of genotypes (e.g. species, cultivars, breeds) and their mixtures, farming practices, exchanges between crop and livestock production, agroecological infrastructures, and the organization of landscapes. Increased diversity can help agricultural systems better adapt to global changes through increased resistance and resilience to environmental fluctuations and extremes (Lin 2011). In addition, it increases the diversity and heterogeneity of agricultural products that will have to be processed and turned into food products (Chable et al. 2020; Prache et al. 2021). In this integrative vision, agroecology can expand only with a new demand for food consumption and only when it is integrated and organized in sectors and territories, as emphasized by some authors (Francis et al. 2003; Gliessman 2006). This extended definition of “agroecology as a food system” has made good inroads into the scientific community (Mason et al. 2021). It has been adopted by INRAE, and thus, its research community now faces an entire chain of expectations and required articulations.

Because this strong agroecology requires deeply transforming agricultural production systems, it will require a major and long-term commitment from agronomic research and articulation with concerned actors of agri-food chains and territories. Such in-depth redesign is a long adaptive process that must be undertaken “on the fly” depending on different spatial and temporal dimensions, along a trajectory that cannot be predefined. The transition phase appears as a research topic in itself (Tittonell 2020). It can temporarily make systems more vulnerable, while aiming at increasing their diversity, resilience, and adaptation to environments and societal expectations.

### Using ecological and socio-economic concepts for renewed research frameworks

From an ecological viewpoint, the agroecological transition is the move from a paradigm based on placing the “ideal” organism in an environment that has been made optimal — the basis of current agricultural systems — to a paradigm based on diversity and interactions between organisms and their integration into ecosystems. Experimental results strongly suggest that increasing the diversity of interacting individuals, varieties/breeds, or species improves adaption to heterogeneous and changing environments (Barot et al. 2017), but this result must still be demonstrated under operational conditions (Table 1). Doing so calls for research that focuses on biotic interactions and ecosystem functions and services, by applying functional ecology concepts to agroecosystems (Garnier and Navas 2012). Examples of such concepts include (i) stoichiometry, to identify plant and animal associations that can take advantage of nutrient proportions, synergize various needs and availabilities, and introduce recycled resources (Bertrand et al. 2019); (ii) phenotypic plasticity of species, to understand and use the adaptation of species to varied and/or fluctuating environmental conditions (Nicotra et al. 2010); and (iii) trophic and mutualistic networks, to leverage connections between species, whether food (trophic) connections or relationships that have reciprocal (mutualist) benefits such as symbioses (Bascompte 2010; Pocock et al. 2012). The corresponding research will help to identify the arrangements of varieties/breeds and species that best provide agricultural production and other ecosystem services and will help to provide insights into how to manage them sustainably.

**Table 1:** Using ecological, socio-technical and socio-economic concepts for renewed and tested research frameworks (adapted from Caquet et al. 2020). The first column describes ecological objectives on which agroecology is based compared to conventional agriculture. The second column describes socio-technical and socio-economic consequences for innovation and scaling up of agroecology compared to conventional agriculture.

**Objectives**		**Consequences for agricultural development**	
**Conventional agriculture**			
**Individual-focused paradigm** Obtaining the highest-performing individual in an optimal environment		**Norms and references** Producing and adapting norms and references in agronomy	
**Agroecology**			
**Adapt and use ecological concepts in agroecosystems**		**Emergence of innovative socio-technical niches and conditions for scaling them up**	
Reconsider living organisms ➔ interactions Stoichiometry Phenotypic plasticity, adaptation, evolution, etc.	Reconsider diversity ➔ integration at field, farm and landscape scales Trophic and mutualist networks, spatial ecology, etc.	Socio-technical conditions for the agroecological transition New cropping systems, decrease in nutrient and pesticide inputs, crop-animal integration, etc.	Socio-economic conditions for transitions of agri-food systems New agricultural products, new processing chains and markets, working time, etc.
**Design agroecosystems** **from field to landscape scales**		**Transitions and breaks with the status quo** **in territories and agri-food systems**	
Exploration of resources (symbioses, mixtures, etc.), recycling		Functional integrity of the agri-food system and territories	
**Paradigm of interaction and integration** Obtaining the highest-performing combinations in heterogeneous and changing environments		**Sharing of learning experiences** Adaptation loops, new equipment	**Values for system success** Including ecosystem services, and social and economic dimensions

From a socio-economic point of view, the agroecological transition aims at moving from a paradigm based mainly on product and production standards, applying them everywhere in all circumstances and adjusting them roughly through inputs (e.g., water, pesticides, nutrients), to an alternative paradigm based on adapting agricultural systems to local conditions (e.g., requiring much more short- and long-term information on the farm, territory, and food chain); encompassing environmental, social, and economic dimensions; and aggregating them to increase adaptability (Table 1). Vulnerability and resilience are new topics for agroecological research, especially in contexts of climate change, health crises, and price volatility. The vulnerability of agroecosystems to shocks, which used to be compensated for by short-term use of inputs, is now considered over the long term. Resilience, defined as the capacity to absorb disruptions and to reorganize while undergoing change (Walker et al. 2004), is a major emergent property that is related to higher diversity in agroecosystems, but also to enhancing the education and adaptability of actors (Leippert et al. 2020; Sinclair et al. 2019). Several socio-economic concepts must be used to do so: adaptiveness (Darnhofer et al. 2010), learning by doing and experimental doing (Kolb and Kolb 2009), step-by-step design (Meynard et al. 2012), and sharing of experiences and living labs (McPhee et al. 2021). Dedicated indicators will have to support this transition of actors, producers, and consumers, based on values related to ecosystem services and human dimensions that are assumed, recognized, and even fostered in agri-food chains and territories.

## Six issue-specific themes

### The genetics mechanism and its implications for animal and plant breeding

Agroecology studies often stop at only one taxonomic level: species diversity and interactions between species. Considering the contribution of intraspecific genetic diversity remains a challenge. Extending genetic diversity locally can contribute to the performance of agroecological systems. Doing so requires deeper understanding of the underlying mechanisms, including their interactions with the environment, and identifying the major traits of interest involved in interactions between plants, between animals, and between plants, animals and their environment. Improving identification of the range of diversity that allows for the expression of mechanisms that yield more resilient production systems is also a subject of interest. A few pioneer studies, either experimental or model-based, have shown that genetic diversity could increase the resilience of plants or animals to environmental changes (Hughes and Stachowicz 2004; Muchová and Fazekašová, 2010) or epidemics (Springbett et al. 2003). Promoting genetic diversity within crops has only recently been included in a global approach to evolutionary agroecology (Mercer 2018).

Breeding strategies will have to evolve to consider objectives of diversity (Litrico and Violle 2015), not only at the individual level (plasticity), but also at the level of the group of individuals that make up the plant cover or the herd, and to define strategies for using diversity based on the ecosystem services desired in a given environmental context. These strategies will have to be included in the management of agricultural systems. In some situations, genetic diversification can lead to more work for farmers or breeders, increase the risk of inconsistent product quality, and thus, challenge the ability to automate processing. Genetic diversification must thus be assessed over the entire agri-food chain, from the production unit to the agricultural and food product. Three main lines of research have been highlighted:

- Understanding and estimating the genetic aspect of interactions in complex crop stands (Bolnick et al. 2011; Meilhac et al. 2020) and developing breeding methods that steer or take advantage of this genetic variability (Sampoux et al. 2020), something that is not currently done in monospecific and monovarietal systems. The trait-based approach can include a functional aspect and opens the potential to obtain breeding criteria for plants and animals.
- Studying relationships between the genetic diversity of crops (Hajjar et al. 2008) and/or livestock and agroecosystem services in order to analyze which services the genetic mechanism can improve, considering its use in heterogeneous environments in which genetic-environment interactions play a central role (Barot et al. 2017). Which plant and animal traits should or should not be diversified to optimize certain ecosystem services?
- Studying the genetics in a wide range of G × M × E × C × P interactions (G: genetic variability between individuals and between populations; M: variability in the microbial environment (e.g., intestinal microbiota; microbiota of the leaf, root, or soil); E: variability in the environment (e.g., climate, water resources, soil type) in connection with agroecosystem functioning; C: management of the farm, production system and cropping system; P: product variability).

These challenges of leveraging genetic diversity for agroecology, which are similar for animals and plants, call for a renewal of genetic research. This renewal can aim to (i) use the genetic mechanism more often in all systems approaches, whether in agronomy or economics; (ii) supplement experiments with high-resolution phenotyping, through participatory work with farmers and breeders, to increase the diversity of situations studied (e.g., farm networks, experiments co-designed with actors); and (iii) develop breeding strategies, co-constructed with actors, for mixed-breed or mixed-line populations or for crossbreeding. This genetic mechanism will benefit from participatory research to help identify, in a variety of local contexts, relationships among genetic characteristics, the environment, and the quality of final products.

### The landscape mechanism to contribute to agroecosystem services

The landscape is a major mechanism of agroecology due to its structuring elements (e.g., “green infrastructure” such as hedgerows and woods) and spatio-temporal organization of crop rotations, grasslands, and cropping and livestock practices (i.e., “landscape of practices”). Landscape characteristics influence biodiversity and major associated ecosystem services such as pollination and the regulation of pests, weeds, and diseases (e.g., Karp et al. 2018). The landscape also contributes to the regulation of water, soil, and air quality and the maintenance of plant and animal production (e.g., soil fertility, animal nutrition). It combines management scales (e.g., field, farm, territory, watershed) and ecological scales (from the square centimeter to the agricultural landscape) that take part in a double environmental and social history (Gascuel-Odoux and Magda 2015).

Knowledge is required on compositions, spatial arrangements, and types of management for multifunctional and resilient landscapes, and the coexistence and complementarity of production systems in a territory. The issue of social trade-offs between ecosystem services, their determinants, and consequences on natural resource management are expanding areas of research. It is a matter of finding public policy instruments that can promote methods to coordinate management and to identify the actors required and modes of governance to implement these instruments (White et al. 2012). The goal is to make progress in the design of multifunctional and resilient landscapes. Four corresponding lines of research have been highlighted:

- Analyzing effects of heterogeneity at multiple scales of space and time. Doing so involves understanding interactions between abiotic and biotic components and between biogeochemical and ecological ones. It further requires understanding the dynamics of populations (e.g., disease vectors), interacting populations (e.g., pathosystems), and communities (e.g., natural enemies of pests) in heterogeneous and changing environments. The influence of interfaces between cultivated and semi-natural environments (e.g., refuge, exchange of organisms) remains poorly documented, whether for disease management (Alexander et al. 2014), biological control, or pollination. Little is known about landscape-scale effects of agricultural diversification on the intensity and stability of ecological processes of interest in agroecology (Petit et al. 2020).
- Developing integrated approaches to address the multifunctionality of landscapes. Abiotic processes are usually considered in a compartmentalized manner, by chemical element or landscape object, and are studied separately from biotic processes (Vinatier et al. 2016). Few ecological processes have been considered simultaneously. Studies of a set of flows in an integrative vision remain rare; for example, the ability to analyze the coexistence and interactions of different production systems in the same territory remains limited.
- Identifying and associating the diversity of landscape actors through research into public policy instruments, more voluntary instruments such as non-economic incentives (e.g., nudges) and processes of common framing and collective learning (e.g., community building) among landscape actors. Identifying incentive mechanisms that can help coordinate actors in a territory to promote agroecological management of the landscape remains a major challenge.
- Supporting the design of agroecological landscapes by mobilizing actors (e.g., farmer groups, cooperatives, NGOs, local authorities), developing indicators and decision-making tools, and leveraging participatory or action-research mechanisms (Berthet et al. 2016). Modeling and simulating biophysical and ecological processes, identifying decision rules, and playing serious games can contribute to the design of agroecological landscapes by explicitly considering the demands and organization of actors.

The research community faces a lack of observation datasets that are coherent, well distributed spatially and that span long periods. Therefore, to design or analyze agroecological landscape systems, several complementary priorities must be addressed: (i) establish interdisciplinary observation and/or experimentation sites (experimental landscape ecology, e.g., Allan et al. 2015), such as LTER sites; (ii) improve observation tools, in particular through new sensors (i.e., proximity and remote sensing) to obtain high-density data at high frequency for all variables (i.e., biotic, abiotic, and actor practices) needed to analyze ecological and agronomic phenomena; (iii) improve statistical approaches in spatial ecology (e.g., regression, decision tree, spatial statistics) and their extension to the spatio-temporal domain; and (iv) implement participatory approaches more widely to complement scientific observation with the actors’ expert knowledge or with non-formal observations recorded by non-scientific and non-professional actors.

### Agricultural equipment and digital technology to better monitor biological systems

Agricultural equipment and digital technology are developing rapidly and have the potential to accelerate the industrialization of agriculture. Using them to support the agroecological transition must therefore be specifically thought out in order to develop tools to leverage differences environments and reduce the costs and risks associated with the agroecological transition (Gunnarsson and Hansson 2004; Salembier et al. 2020) while keeping the initial function of mechanization on easing the work of farmers and implementing management practices, which change from conventional to agroecological systems. The literature rarely addresses these subjects (Bellon-Maurel and Huyghe 2017; Cupial and Kowalczyk 2020; Machenaud et al. 2014). In particular, suitable mechanization appears to be a black box of the agroecological transition literature, except in countries of the Southern Hemisphere (Baudron et al. 2019). Societal initiatives such as “peasant workshops” promote open innovations in machinery help to fill this gap (Chance and Meyer 2017). However, precision agriculture and precision livestock farming, which refer more to weak agroecology, are well documented.

Digital technology can help provide monitoring and support tools and assist in the adoption of agroecological practices through high-resolution monitoring of the dynamics of environments and agroecosystems. This requires acquiring information and having the associated processing capacity to monitor flows. Five lines of research have been identified:

- Advancing means to measure local biodiversity in connection with the local environment (Johnson et al. 2010). Developing continuous phenotyping of crops, livestock, and major components of the environment will aid agroecological management. Considering effects of biotic interactions on the phenotypic value will allow for appropriate monitoring of systems.
- Using sensors and mapping tools, both off-line and onboard (installed on agricultural equipment), more often to adapt crop management to the heterogeneity of environments (e.g., selective weeding of crops).
- Fostering technologies (e.g., densimetric, optical) to assess product heterogeneity better, both upstream and downstream of the farm. Two key moments are of interest: prophylactic operations before sowing of farm-saved seeds and post-harvest sorting to achieve market-required homogeneity.
- Developing methods to quantify ecosystem services to determine payments for providing them and to assess compliance with certified production methods. Areas of interest include adapting multi-criteria evaluation methods and internalizing externalities into product prices. Agricultural equipment and digital technology can be used to assist in monitoring, and in estimating insurance coverage of risks.
- Equipping the “perception-action” loop with instruments to connect assessment to action (Gaussier et al. 1998). Multiple scientific fields must be used to help make measurements, interpret them, make decisions and perform actions.

Because agroecological processes are inherently dynamic, it is necessary to acquire and use suitable data to monitor trajectories. Several new technologies are useful in this respect, ranging from high-resolution remote sensing to in situ sensors that can characterize changes in the environment and its heterogeneities, and monitor trajectories of major biological processes that influence yields, looping of biogeochemical cycles, and regulation of water resources (Bauer et al. 2019). This process will generate large amounts of data, and high-performance tools will be required to incorporate them into monitoring and decision support systems. The issues of data access and of rights and obligations for their use, reuse, and dissemination may require legal expertise and must be anticipated in order not to undermine future technological developments.

Research is expected in two distinct fields: “phenotyping tools” and “monitoring and management tools.” Methods to capture interactions among genetics, the environment, and agricultural management constitute a new area of research.

### Modeling to understand and predict dynamics of new agroecosystems

Modeling can help represent relations between agricultural systems and practices, rural landscapes, biodiversity, functions, and ecosystem services of agroecosystems. Although knowledge gaps and the many complex ecological interactions in uncertain environments make modeling a challenge, it is expected to guide and accompany the transition of agricultural systems. It can help understand dynamics of agroecosystems, orient actions and estimate risks and gains of from the agroecological transition. Five lines of research have been identified to meet the challenge of modeling in agroecology:

- Address knowledge gaps on interactions among structural or functional components of agroecosystems. Currently, agronomic models consider few ecological interactions. For example, few multi-species models of forests, grasslands or weeds consider competition (e.g., Dufour-Kowalski et al. 2012). Little research has been done in agronomy on interactions between belowground and aboveground biodiversity, between biotic and abiotic processes in landscapes (Vinatier et al. 2016), or the dynamics of food webs. Increasingly comprehensive knowledge of microbial communities and their functions offers opportunities to represent them better in models of soil biogeochemical cycles (Moorhead et al. 2014). The next challenge is to go beyond the microbial level and consider soil fauna as well (Grandy et al. 2016).
- Develop different modeling approaches to complexity. They include stochastic approaches that consider weak effects by adding a noise term; introducing constraints that decrease data requirements, as in mechanistic-statistical approaches (Dusseux et al. 2015; Soubeyrand 2016); incorporating data from complex interactions, such as remote sensing data; and network theory (Dee et al. 2017). Expectations of learning methods are high. They constitute powerful tools for analyzing “big data” and show promising results in agriculture (Kamilaris and Prenafeta-Boldu 2018). New computational developments have been made in exploring simulation results through data mining, learning methods, visualization, and querying of spatio-temporal data (Bouadi et al. 2017). The concept of risk goes hand in hand with that of adaptive management, adapted as states, constraints, and risk assessment. The concept of resilience is also considered more widely (e.g., Sabatier et al. 2017).
- Consider spatial and temporal dimensions. Spatio-temporal approaches are already used to address a variety of plant and animal health issues (e.g., Hoch et al. 2010; Parisey et al. 2016), biological invasions, or the regulation of chemical and biological flows. Models that capture transient dynamics are essential to study systems not at equilibrium, as in the context of agroecological transition. Spatial patterns often serve as driving variables when models need to predict consequences of spatio-temporal strategies. Spatially explicit approaches are preferred when critical processes interact within a local neighborhood (Turner and Gardner 2015).
- Include phenotypic dimensions to study mechanisms involved in mixture performances (Borg et al. 2018). Dynamic systems that include phenotypic dimensions can already assess effects of selection and migration on the adaptation of a pathogen in a two-host system (Debarre et al. 2013) and interactions with environmental heterogeneity (Papaix et al. 2013). More complex phenotype–fitness landscapes are required to assess mixtures with several varieties, which can experience non-trivial G × E interactions (Hamel et al. 2021).
- Make progress in representing the cascade that connects cropping practices to biodiversity, then to ecosystem functions and services and ultimately to benefits and values (Potschin-Young et al. 2018). Many models explicitly represent biodiversity via traits or ecological guilds, with associated functions, but few of these models are multiservice agroecosystem models, as in any ecosystems in general (IPBES 2016; Lavorel et al. 2017). Multi-criteria approaches for assessing agricultural systems, such as life cycle assessment and decision trees, should consider biodiversity and biological processes better (Sadok et al. 2009; Teillard et al. 2016). Besides ecological models, socio-ecological models that include findings of actors’ behaviors from the human and social sciences should be designed.

Modeling is constrained by the lack of data on the variety of soil, climatic, and agronomic conditions. It is necessary to make better use of observational or experimental programs and to improve them (Bretagnolle et al. 2018). It is also necessary to rely on information from society. The large amounts of data on organic farming systems can be useful if they become accessible, as shown by meta-analyses of the comparative performance of organic and conventional systems (Mondelaers et al. 2009; Tuck et al. 2014).

### The agroecological transition of the farm as a research topic

To date, little research has focused on the agroecological transition of the farm itself (Plateau et al. 2021; Teixeira et al. 2018); however, this scale is key for completely redesigning production systems (Duru et al. 2015a). In this transition, farmers must abandon the vision of a planned production system supplied with inputs that delivers outputs and move toward a more circular agroecosystem functioning that delivers agricultural goods among other services (Tittonell 2013). This transition requires a long-term commitment; removal of technical, organizational, cognitive, and ideological lock-ins; redefinition of performance and other professional norms; adoption of co-design methods with farmers and transformation by trial and error managed through path-finding assessment methods.

- Designing an agroecological production system requires that advisory structures modify their postures, no longer specifying what actions to take but instead supporting the actors involved by obtaining or producing the knowledge necessary to guide them. Three lines of research have been identified (Martin et al. 2018):
- Modeling a farm to explore the field of possibilities. A farm is a complex system managed according to cognitive structures (e.g., goals, plans, preferences) that evolve over time and influence the farmer’s decision-making processes in the context of particular physical (field), social (farm groups), and organizational (labor) structures. Therefore, multiple management elements must be considered to address the trial and error that farmers practice and expected properties such as resilience, risk and viability. To promote interaction with field actors, static models, and parsimonious meta-models may be preferred to dynamic, complex, and time-consuming models. Different types of information can be obtained through serious games, which constitute a medium for knowledge sharing and learning (Stanitsas et al. 2019). The actor network constitutes an important element of the agroecological transition since it allows for the incorporation of collaborative dimensions, exchange of products (e.g., fodder, manure), and sharing of resources (e.g., machines, labor).
- Analyzing the agroecological transition of farms while it happens. First, methods need to be developed to identify systems in transition, thus constituting a “hunt” for innovations, and to understand their potential upscaling (Salembier et al. 2016). Second, concepts and methods need to be developed to investigate dynamics of systems — especially resilience, vulnerability, efficiency, and viability (Bouttes et al. 2018) — and the transformations of work (e.g., organization of activities, changes in the mental workload required to manage complexity, and uncertainty, transformation of the professional worlds of reference) (Coquil et al. 2017). Third, it is necessary to characterize management elements involved in the transition (e.g., management of plant and animal health, biodiversity, integration of activities in diversified systems) and determine how to reconfigure them.
- Designing methods to support the agroecological transition of farms. The field of participatory research involving field actors, especially farmers and their advisors (Lacombe et al. 2018), must be given priority. It is necessary to (i) create design methods to propose locally adapted solutions (Cerf et al. 2012); (ii) develop project management and governance methods that encourage participation, cross-learning, sharing of experiences and scientific investigation in action, and a relationship between the collective and the individual (Van Poeck et al. 2020); (iii) promote learning based on research, advice and training by sharing various modes of investigation; and (iv) analyze and propose management indicators and tactical and strategic reasoning to help farmers reduce the complexity of managing agroecosystems. System experiments in farm research units are also interesting approaches for step-by-step design of agroecological systems, especially when they include actors as co-designers (Bos et al. 2009; Lefevre et al. 2020).

Transdisciplinary approaches must be strengthened to develop the practice of joint work by researchers and non-researchers (Hazard et al. 2020). Obstacles lie in both the research community and field actors due to society’s longstanding perception of science as a top-down mechanism to provide answers to society’s problems. The idea is to consolidate investigation methods and reflective practices that mixed communities of researchers and field actors can use and to succeed in combining the innovation with the academic requirements of scientific knowledge production (Newig et al. 2019).

### Integration of agroecology into food systems

Groups of commodities in existing food systems are organized along value chains (Abecassis et al. 2018; FAO 2014). The way these chains currently operate is considered unsustainable because of the overexploitation of resources, over-consumption and avoidable waste streams, unhealthy diets, and food and nutritional insecurity (Willett et al. 2019). Consequently, transitioning from linear “make-use-dispose” chains to fair, just, circular, and sustainable food — and bioeconomic — systems has been recommended (SAPEA 2020). These ideas seem to be fully in line with the agroecological transition (de Vries et al. 2021). Agroecology focuses in particular on the diversity of resources, closed cycles of organic matter (de Boer and van Ittersum 2018), changes in participatory organization models (Chiffoleau 2019), and labels for protecting local products (Bonroy and Constantatos 2015). Consequently, major elements of food systems must be reconsidered substantially, which poses many new research questions. Thus, lessons learned from elements such as organic food chains (Barabanova and Moeskops 2019; Lacour et al. 2018), traditional food programs (Cotillon et al. 2013), new eco-friendly processing methods closer to production sites (Bruins and Sanders 2012; De Vries et al. 2018), alternative (agri-)business models (Donner et al. 2020), revamping of labels (Asioli et al. 2017), and existing scenario models such as AgriMonde Terra (Aubert et al., 2019; Le Mouël et al. 2018; Mora et al. 2020) allow these questions to be addressed in a structured way. Four lines of research have been identified, each of which raises new questions:

- Products from agroecological farming models. How do their nutritional and functional properties differ from those of products from conventional production models, and how should they be monitored? Should separate value chains be created in which properties as well as production and territorial characteristics are recognizably leveraged in economic, environmental, and social terms (Abecassis et al. 2018), possibly bringing the location and scale of processing closer to production (Bruins and Sanders 2012; De Vries et al. 2018)? These questions also concern the co-products of agricultural resources and the recycling of partially used (food) products. This implies completely revisiting the sustainable use of resources as well as principles that are valid for both agroecological production and food and bioeconomic systems.
- Consumer behavior. Are consumers willing to pay for agroecological products, and if so, would their buying criteria include environmental factors, biodiversity, and locality? How should products be marketed? Will consumers be willing to participate actively in the development of agroecology projects? These questions call for new scientific insights at the interfaces between consumer, social and communication sciences, and at those between agroecological and sustainable food system sciences. This may then lead to revamping of labels; certification programs; marketing strategies and modes of communication; training and participatory programs; and production, processing, and distribution practices.
- Organization of markets, standards, and public policies. Certification in organic farming is a tool to leverage its practices. Should other labels related to agroecology require certification? Initiatives in this regard are being taken locally. Agroecological practices could play a central role in the European Union Common Agricultural Policy (CAP) and its “greening”, through cross-compliance, agri-environmental and climate measures, and more generally, through payments for ecosystem services.
- The territorial and international dimensions of the organization of markets. Can agroecological practices lead to reorganization of agri-food chains and markets? What are potential implications of reorganization at the national, European, and international levels? Of particular concern is the concept of “leakage”, in which relocating a type of production that emits large amounts of greenhouse gases to countries with less restrictive legislation ultimately leads to an overall increase in its emissions.

These questions call for methodological work on (i) data acquisition, management, and analysis from the microscale (e.g., individual, product, company) to macroscale (e.g., regions, countries, large world regions); (ii) modeling agri-food systems to estimate their environmental impacts throughout the entire chain and understand repercussions of local changes on large regions (Gasselin and Hostiou 2021; Kolb and Kolb 2009; Le Mouël et al. 2018); and (iii) case studies to fully understand the diversity of food systems and their constraints. Establishing and managing territorial platforms and experimentation centers can serve as a basis for participatory activities that mobilize a variety of actors to span the chain from production to consumption.

## Issues common to all six themes and next steps

The interdisciplinary assembling of knowledge that characterizes agroecology now appears to be a self-evident dimension. It is a matter of training “assemblers” of skills in order to promote a vision at the interfaces between agriculture, the environment and food systems, and at those between biotechnical and social sciences. The analysis (Table 2) reveals transversal issues among the six themes analyzed, which can be divided into three main categories.

**Table 2:** Main research-issue-specific themes and sub-themes specific to agroecology, from a prospective exercise performed at INRAE. (G: genetic, M: microbial, E: environment, C: cropping, P: product).

Issue-specific themes	Sub-themes	Common issues
The genetic mechanism and its implications for animal and plant breeding	• Steering or taking advantage of genetic variability. Breeding criteria for plants and animals. • Assessing effects of genetic diversity (crops and livestock) and agroecosystem services • Developing genetics in a wide range of G × M × E × C × P interactions	• Collecting biological data to redesign agroecosystems in a participatory approach • Adaptability and risks through a transformation process • Conditions for scaling up: performance and resilience assessment, social learning, socio-economic viewpoints
The landscape mechanism to contribute to agroecosystem services	• Assessing effects of heterogeneity at multiple scales of space and time • Developing integrated approaches to address the multifunctionality of landscapes • Identifying and associating the diversity of landscape actors • Supporting the design of agroecological landscapes by mobilizing actors	
Agricultural equipment and digital technology to better monitor biological systems	• Advancing the means to measure local biodiversity in connection with the local environment • Fostering technologies to assess product heterogeneity • Developing methods to quantify ecosystem services and potentially to determine payments • Equipping the “perception-action” loop with instruments	
Modeling to understand and predict dynamics of new agroecosystems	• Making advances in key knowledge gaps regarding interactions among agroecosystem components • Considering spatial, but also temporal, dimensions in models • Including phenotypic dimensions in agroecosystem performances • Representing the cascade from practices to biodiversity, ecosystem services, benefits, and values	
The agroecological transition of farms as a research topic	• Modeling farms to explore possibilities (e.g., cognitive, environmental, social, organizational…). • Analyzing the agroecological transition of farms as it happens (data, obstacles and mechanisms) • Designing methods to support the agroecological transition (participatory research approach)	
Integration of agroecology into food systems	• Characterizing products of agroecological systems • Characterizing consumers’ behavior and willingness to support agroecological products • Characterizing organization of markets, standards and public policies, and the place of agroecology • Effects of territorial and international organization of markets for agroecological products	
Next steps: relevance, implementation, and funding		

### From collecting biological data to using them to redesign agroecosystems in participatory mechanisms and transdisciplinary approaches

In industrialized countries, agroecology must contend with a certain scarcity of data on systems in transition and on agroecology-based systems. Digital agriculture, proximity and remote sensing methods, and spatio-temporal data analysis can help fill this gap. Experimental mechanisms can focus even more on agroecology, but other sources of data, such as farm networks, must also be used (Sumane et al. 2018). Reflection is needed on how to better collect, freely provide, and use data that are currently scattered, incomplete, and excessively centered on production systems at the expense of upstream and downstream sectors.

More research should be conducted to leverage knowledge developed within actor networks created through “actor-driven” or “citizen-driven” societal initiatives (e.g., Van Etten et al. 2019). “Open innovation” (Von Hippel 2016), a process in which a variety of actors co-create and contribute to innovation through adapted governance, must be developed, especially by living labs within territorial projects or agri-food chains (McPhee et al. 2021). Such mechanisms foster dynamics for exchanging knowledge, know-how, and experience between actors. Transdisciplinarity can accelerate innovations for the agroecological transition, and research should become more involved in these dynamics for all six research topics.

### Understanding adaptability and risks though a transformation process

The expectation of the agroecological transition is that a decrease in input-based gross productivity will compensated by improvements in health and the environment, and greater resilience to shocks. In the short term, the transition may represent a risk to farmers (Tittonell 2014) and probably even more so to agri-food chains, which will have to manage a greater variety of actors, products, and uncertainties. Agroecology-based systems will depend more on neighborhood effects, landscape elements, and the functioning of metapopulations.

Exchanges will increase and will require coordinating all actors of the agricultural collective (e.g., cooperatives) and of processing industries in territories engaged in the agroecological transition. Management of agricultural and agri-food activities will no longer be “predetermined” but must be open-ended, adaptive, and creative, with objectives and decisions being adapted to the available information, in both the short and long terms, to better anticipate risks (Lamine et al. 2021). In this respect, the COVID-19 crisis can act as a shock and impetus to accelerate the development of agroecology, with a reterritorialization of food production and processing.

### Scaling up

This transition is possible only if it engages consumers (e.g., individual purchases, collective catering), citizens, and public authorities, whether European (CAP reform), national (e.g. support of the organic-production chain, changes in education programs), or local (e.g. local food plan, school canteens), all of which contribute to innovations and regulations. Four approaches seem to be essential to support broad-based transitions and scaling up: (i) performance assessments that emphasize resilience to hazards, coverage of ecosystem services, and gains in sustainability in environmental, social, and economic dimensions (Tittonell 2020); (ii) modeling, to better predict the evolution of agricultural systems at different times and over different periods, better manage new agroecosystems, and help identify dead ends, risks, and capacities for robustness and resilience (e.g., Padro et al. 2020); (iii) social learning, through sharing experiences and knowledge of various origins (Gimenez Cacho et al. 2018) in a participatory-science approach (Mendez et al. 2017) to broaden the spectrum of innovations and their testing under different conditions; and (iv) analysis of implications and conditions as mechanisms for improvement, such as work time (Duval et al. 2021), analysis of the perception and objectification of risk, and its support from a socio-economic viewpoint. These research approaches will enrich how agricultural development advice is conceived. The agroecological transition will rely not only on prescriptive solutions, but on frameworks and tools co-constructed with the research community to promote the adaptive capacity of actors.

### Next steps: relevance of the research agenda, implementation, and funding research for agroecology

Scientific organizations and program managers of research-funding bodies can take advantage of INRAE's exercise by using and testing the method developed here and by discussing the topics identified according to their own contexts (e.g., region, country, agri-food sector). To our knowledge, this kind of approach has never been used. Its relevance can nevertheless be measured within INRAE by its incorporation into the agendas of INRAE’s scientific divisions, for INRAE researchers by their many publications on agroecology since 2014 and at the European level by the recognition of INRAE’s expertise in agroecology in Europe. For example, some of this strategy has been incorporated into the European Union–funded coordination and support action “ALL-Ready”, which is based on open innovation arrangements, especially living labs and research infrastructures, as instruments to scale up agroecology in Europe.

This exercise highlighted the multiple mechanisms (e.g., genetics, landscapes, agricultural equipment, digital technology) that can be used to promote agroecology. Since they can be applied to all agricultural systems, we emphasize their importance and specific orientation for agroecology. We also stress the importance of integrating agroecology at multiple levels in the production chain, such as farms and food systems, and of collecting data to redesign agroecosystems using participatory approaches (Atta-Krah et al., 2021). Such a vision can lead to new strategies for funding research and innovation in agroecology, particularly at the local scale (e.g., territories), through funding from the European Union, regions and large cities, sometimes with agricultural and agro-industrial partners. This has happened recently, notably in France through “innovation territories”, which often include living labs funded by large consortiums for agroecology.

Methods should now be developed to test the relevance of this agenda with agricultural (e.g. farmers, advisors, companies) and non-agricultural actors (e.g., NGOs, policymakers). Actors have been exposed to the agenda in 1-day events, such as a presentation followed by round tables or discussions with actors who sit on INRAE boards. The main steps of this process can include the following guidelines: (i) map the initiatives in which research bodies are involved at national and European levels, and characterize them (e.g., topics, co-building, contexts); (ii) map key actors at national and European levels; and (iii) survey research agendas to identify what actions are underway, what is needed, the obstacles to remove, the mechanisms to activate and how to make progress with actors. Knowledge and design tailored to the specific needs and goals of agroecology would thus constitute a valid scientific and societal contribution.

The pattern for the development of agroecology, either as a dominant form for the entire agricultural sector and all agricultural land, for which the agroecological transition will progress step by step depending on the context, or as a specific and multifunctional form of agriculture particularly used by specific sectors or in specific environments is currently under debate. Resolving this debate calls for methods to evaluate agroecological systems, innovations, and trajectories along their transformation pathways.

## Conclusion

This article addresses the construction and dissemination of a research agenda for agroecology, using the example of the approach adopted by a major research institute. This contribution is original because it addresses many dimensions of agroecology. Recently, however, there has been a curious lack of research agendas for agroecology with such a broad scope (but see Tittonell et al. 2020). Its context is the transformation of agriculture in industrialized countries, which have high levels of technological ability (e.g., genetics, agricultural equipment) and excellent data acquisition and modeling abilities. Agriculture in these countries forms part of agri-food chains and territories that must be transformed to scale up agroecology. This research aims less for results in the near future and more for a long-term step-by-step adaptive transformation to move toward a redesign of production methods, based on a variety of mechanisms and the use of ecological, socio-technical, and socio-economic concepts.

Agroecology promotes ecological processes as driving forces and insurance for the resilience and sustainability of agri-food systems that are facing climate change and other crises. Several studies support this assertion. However, questions remain about agroecology’s consequences on the amount of farm work, the reterritorialization of agricultural and food production, and the quantity and quality of food the global scale, especially in the context of climate change and tension over water resources.

While a large-scale agroecological transition of agriculture in industrialized countries is urgently needed to limit its impacts on human health and the environment, the degree of intensity and spatial coverage of agroecological systems remain an open question, as well do the benefits of the coexistence of “green” industrial and agroecological systems for fostering resilience, innovation, and development in food chains and territories. Agronomic research in the broadest sense, including its environmental and food components, will no doubt use this transition as an opportunity to renew itself and contribute to the production of new societal norms.

## Data Availability

Not applicable.
